# Dexmedetomidine exerts neuroprotective effects during high glucose-induced neural injury by inhibiting miR-125b

**DOI:** 10.1042/BSR20200394

**Published:** 2020-06-26

**Authors:** Xiaolai Hou, Fenlan Xu, Cheng Zhang, Jianzhong Shuai, Zhenhua Huang, Yu Liang, Xiaoyan Xu

**Affiliations:** 1 Department of Anesthesiology, Shanxi Provincial People's Hospital, Taiyuan, China; 2 Department of Anesthesiology, The Public Health Clinical Center of Chengdu, Chengdu, China; 3 Department of Anesthesiology, Chengdu Women’s and Children’s Central Hospital, Chengdu, China; 4 Laboratory of Anesthesia and Translational Neuroscience Center, West China Hospital of Sichuan University, Chengdu, China

**Keywords:** Dexmedetomidine, diabetic neuropathy, miR-125b-5p, vitamin D receptor, high glucose

## Abstract

Diabetic neuropathy (DNP) is the most common complication of diabetes mellitus affecting approximately 50% of diabetes patients. Studying the effect of potential drugs with antioxidant properties and minimal toxicities on neural cells may lead to the development of new and safe pharmacotherapy. Dexmedetomidine (DEX), a highly selective α2-adrenoceptor agonist, is a clinically used sedative also known to have neural protection effect. In the present study, we aimed to investigate the protective role of DEX in high glucose (HG)-induced neural injury and its potential miRNA-related mechanisms. Our results showed that DEX exerted neuroprotective effects during high glucose-induced damage to PC12 cells in a dose-dependent manner. DEX restored cell viability and repressed LDH, Caspase-3 activity, ROS production, and cell apoptosis in HG-treated PC12 cells. MiR-125b-5p was significantly up-regulated in PC12 cells upon HG treatment and it was demonstrated as an target for DEX. The neuroprotective effects of DEX on HG-induced cellular injury were reversed through miR-125b-5p overexpression, and vitamin D receptor (VDR) is a direct targeted of the miR-125b-5p. Together, our results indicate that DEX displays neuroprotective effects on PC-12 cells under high glucose through regulating miR-125b-5p/VDR axis. Our findings might raise the possibility of potential therapeutic application of DEX for managing diabetic neuropathy neural injuries.

## Introduction

Although glucose is essential for normal neural activity, excessive glucose induced oxidative stress and neuronal cell injury. Diabetic neuropathy (DNP), a heterogeneous group of disorders in patients with diabetes mellitus, involves damage or impaired function of the autonomic and/or peripheral nervous system [1]. More than 50% of individuals with diabetes are affected by DNP, the most common and troublesome complication of diabetes mellitus, leading to the greatest morbidity and mortality and resulting in a huge economic burden for diabetes care [2]. Elevated serum glucose levels even elevated the risk of Type 2 diabetes (T2DM) and DNP in the mother, and obesity, cardiovascular disease (CVD), and T2DM in the child [3,4]. Despite advances in understanding the etiology, and the significant individual and social burden associated with DNP, its treatment remains unsatisfactory. Many therapies have been the subject of clinical trials for DNP. However, there are currently no FDA-approved therapies for DNP. Therefore, identifying novel therapeutic strategies remains paramount.

Dexmedetomidine (DEX) is a highly selective α_2_-adrenoceptor and is clinically used as a sedative and analgesic [5]. The α_2_-adrenergic receptors are distributed throughout the central and peripheral nervous system [6]. The disturbances of the noradrenergic system, including alterations in the α2-adrenoceptors, are posited to be involved in the pathophysiology of several neural disease [7,8]. DEX exerts anti-inflammatory effects during hypoxia-induced neural injury and reduces the oxidative stress response and inhibits apoptosis, thus playing a potential neuroprotective role [5,9]. Zhong et al. reported that DEX alleviates pain in DNP by inhibiting inflammation and astrocyte activation through suppression of the Wnt 10a/β-catenin signaling pathway in rats [10]. However, the effects of DEX on neural injury in DNP remain unclear.

Vitamin D and its receptor (VDR) regulate multiple factors, and it was expressed in neural systems and involved in neural function [11,12]. The reported biological effects of VDR in the nervous system include the biosynthesis of neurotrophic factors, neurotransmitter and the synthesis of inducible nitric oxide synthase and increase glutathione levels, suggesting its potential neuroprotective and immunomodulatory effects. Bioinformatic analysis identified that the expression of diverse miRNA could be modulated by vitamin D treatment [13]. On the other hand, through complementarity with specific miRNA sequences, miRNAs act primarily to silence VDR expression through either degradation or inhibited translation of target transcripts [14]. In this way, miRNAs can act to fine-tune the transcriptional regulation of VDR expression, which may also play distinct roles in the proliferation, differentiation and function of specific cell types. miRNA regulatory networks may be particularly important for VDR signaling. These results reveal potential pharmacological target roles of VDR for SNP of future research.

MiRNAs are short, non-coding RNAs at 18–24 nucleotides length, which modulate target gene expression by interacting with their 3′-untranslated regions. MiRNAs are involved in various biological phenomena including apoptosis, autophagy, cell proliferation and metabolism [15,16]. It has been widely reported that serum levels of miRNAs are altered in Type 1 diabetes and considered to be a promising disease in diabetes diagnosis [17]. MiRNA-31 was reported to contribute to the high glucose-induced endothelial cell dysfunction [18]. However, limited information is available regarding their role in DNP. MiRNAs also contribute to the pathogenesis of diabetic complications including nephropathy and heart disease [19], and could be novel drug targets for disease therapy. In recent years, miR-125b-5p has been reported play an important role in cell inflammation and apoptosis. MiR-125b-5p was reported to implicated in anesthesia-induced hippocampal apoptosis [20]. However, whether miR-125b-5p and VDR regulate high glucose-induced apoptosis in PC-12 cells remains unclear.

PC12 cells were used in our studies because they assume neuronal phenotype and constitute an established model for studying mechanisms of adverse neuronal effects caused by high glucose levels such as apoptosis and ROS generation. PC12 cells also provide an *in vitro* model of neural cells to study the effect of potential drugs and their mechanisms implicated in SNP under HG condition. In the present study, we aim to examine the effect of DEX by cellular and molecular methods *in vitro* on PC-12 cells, under high-glucose conditions to stimulate DNP and to investigate the underlying mechanism. We assessed miR-125b-5p expression under high-glucose conditions and DEX treatment, and investigated its target mechanism.

## Materials and methods

### Cell culture

PC12 cells (ATCC, Manassas, VA, U.S.A.) were cultured in Dulbecco’s modified Eagle’s medium (DMEM) supplemented with glucose (Gibco, Grand Island, NY, U.S.A.) at different concentrations, 100 mg/ml streptomycin and 100 U/ml penicillin (Gibco), 10% fetal bovine serum at 37°C in 5% CO_2_ incubator (Thermo, Waltham, MA, U.S.A.). The glucose concentration in DMEM was 30 mM and was considered as the normal glucose (Con) and 150 mM was considered as the high glucose (HG). The cells were pretreated by, and cells were co-cultured with or without DEX for 48 h for and co-incubated with DEX and HG (150 mM) for 48 h. Equivalent concentrations of mannitol were used as an osmotic control.

### Quantitative reverse transcription polymerase chain reaction (qRT-PCR) analysis

Total RNA of PC12 cells was obtained using Trizol reagent (Takara, Shiga, Japan). Subsequently, miRNA was reversed to cDNA by miScript Reverse Transcription Kit (QIAGEN, Dusseldorf, Germany). SYBR Green PCR Master Mix (Applied Biosystems, Foster City, CA. U.S.A.) was applied to detect the expression of miR-125b-5p in RT-qPCR. U6 was used as an internal control, and the 2-ΔΔCt method was used to normalize target gene expression levels. Primer sequences were as follows: U6: 5′-CTCGCTTCGGCAGCACA-3′, miR-125b-5p: 5′-TCCCTGAGACCCTAACTTGTGA-3′.

### Western blot analysis

Total cellular proteins were extracted from PC-12 cells, using a protein extraction kit (Santa Cruz Biotechnology, Santa Cruz, CA, U.S.A.), and protein concentrations were determined using a BCA protein assay kit (Beyotime P0012S, Shanghai, China) and separated via sodium dodecyl sulphate polyacrylamide gel electrophoresis (15% resolving gel), after which protein bands were electro-transferred onto PVDF membranes (Millipore, MA, U.S.A.). Thereafter, the membranes were blocked in 5% skimmed milk and incubated at 37°C for 1 h and overnight at 4°C with the following primary antibodies (all obtained from Abcam, Cambridge, U.K.): anti-β-actin (rabbit 1:10,000) and anti-VDR (mouse 1:10,00, ab109234). Thereafter, membranes were incubated with HRP-conjugated secondary antibody (1:20,000, anti-rabbit CWBIO, Beijing, China) for 1 h at 37°C, followed by treatment with ECL (Thermo, Waltham, MA, U.S.A.), and the intensity of protein bands was detected using Image Lab™ Software (Bio-Rad, Hercules, CA, U.S.A.).

### Assessment of intracellular production of reactive oxygen species (ROS)

Intracellular ROS was detected using 2,7-dichloro fluorescein diacetate (DCF-DA), which is oxidized to a fluorescent molecule, DCF, by ROS. After glucose or DEX treatment, the media were replaced with serum-free DMEM. PC12 cells were incubated with 10 μM dichloro-dihydro-fluorescein diacetate (DCFH-DA) at 37°C for 30 min, then fluorescence intensity was evaluated using a live cell imaging system (Olympus, Tokyo, Japan) at excitation and emission wavelength of 485/20 and 528/20 nm, respectively.

### Cell transfection

MiR-125b-5p mimics, miR-125b-5p inhibitors and negative controls (NC) were obtained from RiboBio (Guangzhou, China). The pcDNA-VDR vector was purchased from Santa Cruz Biotechnology. Cell transfection was performed using Lipofectamine2000 (Invitrogen, MA, U.S.A.) in accordance with the manufacturer’s instructions.

### The 3-(4,5-dimethylthiazol-2-yl)-2,5-diphenyltetrazolium bromide (MTT) assay

3-(4,5-dimethylthiazol-2-yl)-2,5-diphenyltetrazolium bromide (MTT) assay was employed to assess the cell viability. Cells at the exponential growth stage were resuspended and inoculated in a 96-well plate at 4 × 10^4^ cells/well. After cells were treated with 20 μl MTT (Sigma; 5 mg/ml) in each well for 4 h. Then, the medium was discarded, and 200 μl dimethyl sulfoxide (Sigma-Aldrich, St Louis. U.S.A.) was added to each well to dissolve the formazan crystals. Absorbance was detected at 450 nm using a spectrophotometer (Bio-Tek Instruments, Winooski, VT, U.S.A.). The experiment was performed in triplicate.

### Flow cytometry analysis for apoptosis

After incubation under different conditions for 48 h, PC12 cells were resuspended and stained using a Annexin V-FITC/PI kit (Becton, Dickinson and Company, Franklin Lakes, NJ, U.S.A.) for 20 min in the dark. Apoptotic cells (FITC-positive and PI negative) were distinguished via the flow cytometry assay (BD Biosciences, San Jose, CA, U.S.A.), and the percentage of the apoptotic cells was analyzed using CELL Quest 3.0 software (BD Biosciences).

### Assessment of lactate dehydrogenase (LDH) activity

Lactate dehydrogenase (LDH) release in the culture medium is an indicator of cellular injury and cell death. LDH activity was detected using a CyQUANT™ LDH Cytotoxicity Assay (Thermo, C20300) according to the manufacturer’s instructions, and the absorbance was measured at 490 nm.

### Caspase-3 activity assay

Capase-3 activity was assessed using a caspase-3 activity assay kit (Cell Signaling, Danvers, MA, U.S.A.) in accordance with the manufacturer’s instructions. The caspase-3 activity was measured by cleavage of the caspase-3 substrate (Ac-DEVD-pNA). PC12 cells were lysed after the indicated treatments and the supernatants were incubated with 10 μl of Ac-DEVD-pNA (2 mM) at 37°C for 2 h. The absorbance was measured at 405 nm.

### Luciferase reporter assays

A cDNA sequence containing an miR-125b-5p binding site or a mutated binding site was cloned into the pmirGLO dual-luciferase vector (Promega, Madison, WI, U.S.A.) as VDR-WT and VDR-Mut. The dual-luciferase vector was co-transfected with miR-125b-5p mimic or NC into PC12 cells for 48 h, using Lipofectamine 2000. Luciferase activity was assessed using the Dual-Luciferase Reporter Assay System (Promega).

### Statistical analysis

Data are presented as mean ± SEM values and analyzed using SPSS21.0 software (SPSS Inc., Chicago, IL, U.S.A.). One-way analysis of variance followed by the Bonferroni test for post hoc analysis was employed in multiple-group comparisons, and *P*<0.05 was considered statistically significant.

## Results

### DEX displayed protective effects on PC12 cells under high-glucose culture conditions

**Figure 1:**
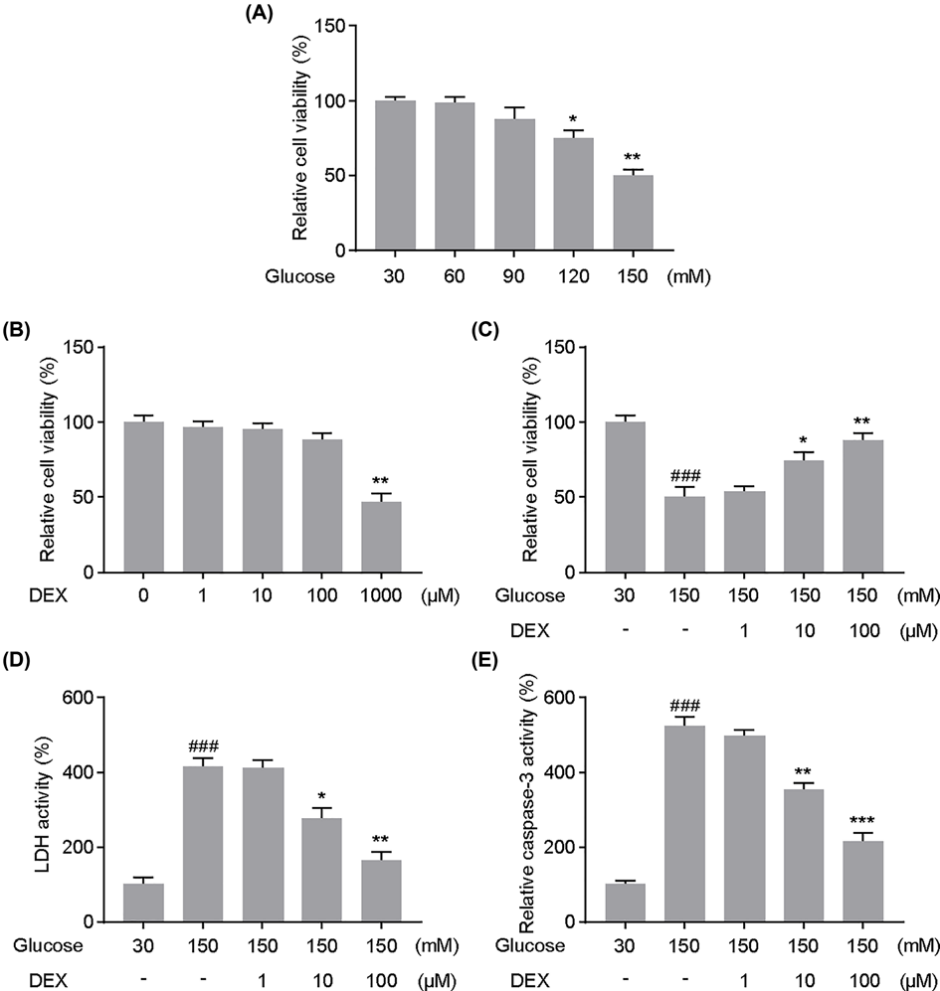
Effect of dexmedetomidine (DEX) on cell survival and apoptosis in PC12 cells under high-glucose conditions The MTT assay was performed to assess the effect of different concentrations of glucose (**A**) and DEX (**B**) on the viability of PC12 cells; *P<0.05, **P<0.01 vs. Glucose, 30 mM or DEX, 0 μM. (**C**) The effects of the DEX on high glucose toxicity of PC12 Cells were assessed by MTT assay. Lactate dehydrogenase (**D**) and Caspase-3 (**E**) activity were assessed to evaluate the neuroprotective effect of DEX in PC12 cells. Cells were co-cultured with HG and DEX for 48 h. ###*P*<0.001 vs. Glucose 30 mM; *P<0.05, **P<0.01, ***P<0.001 vs. Glucose 150 mM. One-way analysis of variance was performed for statistical comparisons. Data are presented as mean ± SEM values, each performed in triplicate.

MTT assay showed that treatment with higher glucose concentrations decreased PC12 cell viability in a concentration-dependent manner (Figure 1A). Equivalent concentrations of mannitol were used as an osmotic control and the results showed that HG had little effect on cell viability (data not shown). The MTT assay was further performed to investigate the effect of DEX on the viability of PC12 cells. DEX at 1,10 and 100 μM did not influence the viability of PC12 cells, while 1000 μM DEX significantly decreased cell viability (Figure 1B). As shown in Figure 1C, PC12 cell viability was significantly decreased upon treatment with 150 mM glucose, while DEX improves the survival of PC12 cells at 10 and 100 μM. LDH and Caspase-3 activity were assessed to evaluate cell injury in PC12 cells. The results indicate that the LDH and caspase-3 activity were markedly increased in PC12 under high-glucose conditions but significantly decreased upon treatment with 10 or 100 μM DEX and under high-glucose conditions (Figure 1D,E). Altogether, these results suggest that DEX attenuates high glucose-induced reduction in survival and apoptosis in PC12 cells.

### DEX decreased ROS production and cell apoptosis in PC12 cells under high-glucose conditions

**Figure 2:**
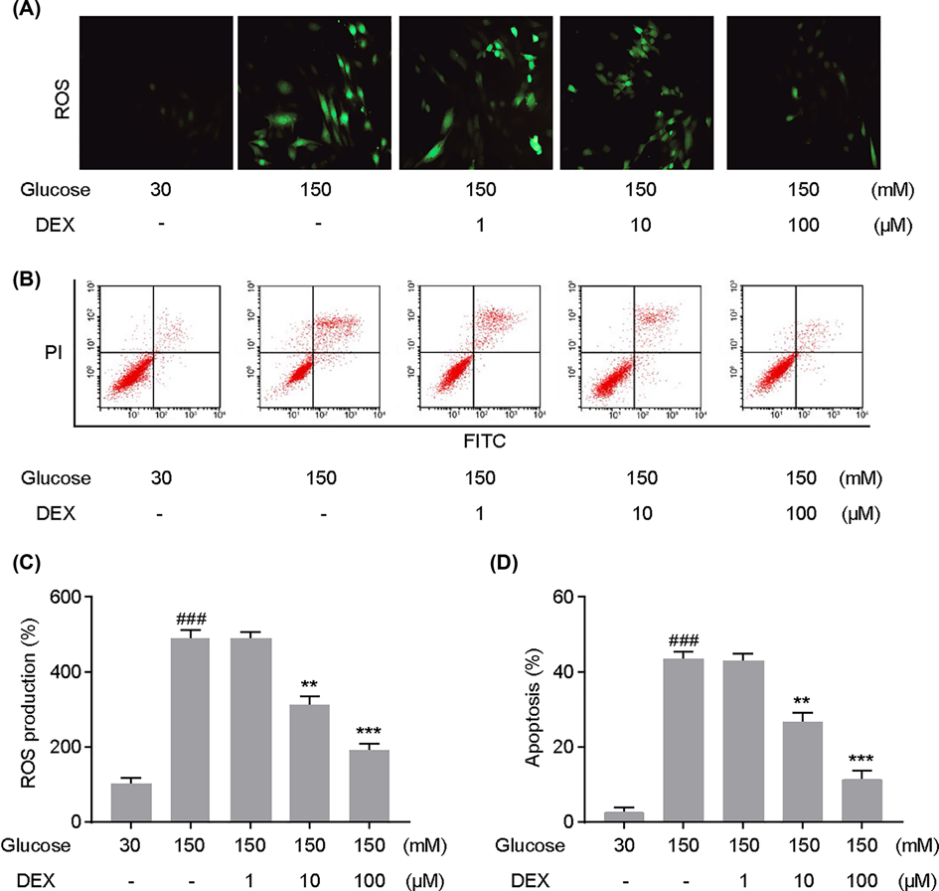
Effects of dexmedetomidine (DEX) on intracellular production of reactive oxygen species (ROS) and apoptosis in PC12 cells under high-glucose conditions Representative images of dichlorofluorescein (DCF) staining (**A**) and the statistics for DCF-fluorescence intensity (**C**). Apoptosis in PC12 cells was evaluated via flow cytometry and using a Annexin-V FITC/PI apoptosis detection kit (**B**) and the statistics regarding the apoptotic rate are shown in (**D**). Cells were co-cultured with 150 mM glucose and DEX (10 or 100 µM) for 48 h. ###*P*<0.001 vs. Glucose 30 mM; **P<0.01, ***P<0.001 vs. Glucose 150 mM. One-way analysis of variance was performed for statistical comparisons. Data are presented as mean ± SEM values, each performed in triplicate.

Previous studies have reported that ROS generation and apoptosis play an important role in high-glucose induced neural cell injury [21]. Therefore, we assessed the effects of DEX on intracellular ROS accumulation in high glucose-cultured PC12 cells, using the DCFH-DA fluorescent probe, which is oxidized to the fluorescent compound DCF by ROS. As shown in Figure 2A,C, DCF-fluorescence intensity significantly increased under high-glucose conditions, while treatment with 10 and 100 μM DEX significantly reversed intracellular ROS accumulation in PC12 cells. The Annexin V-FITC/PI assay was performed to assess the anti-apoptosis effect of DEX. Flow cytometry analysis displayed that high-glucose conditions increased the apoptotic rate in PC12 cells when compared with the control group, while treatment with 10 and 100 µM DEX markedly attenuated high glucose-induced apoptosis in PC12 cells (Figure 2B,D). The present results indicate that DEX treatment inhibited intracellular ROS accumulation and apoptosis in PC12 cells.

### MiR-125b-5p was up-regulated in PC12 cells under high-glucose conditions

**Figure 3:**
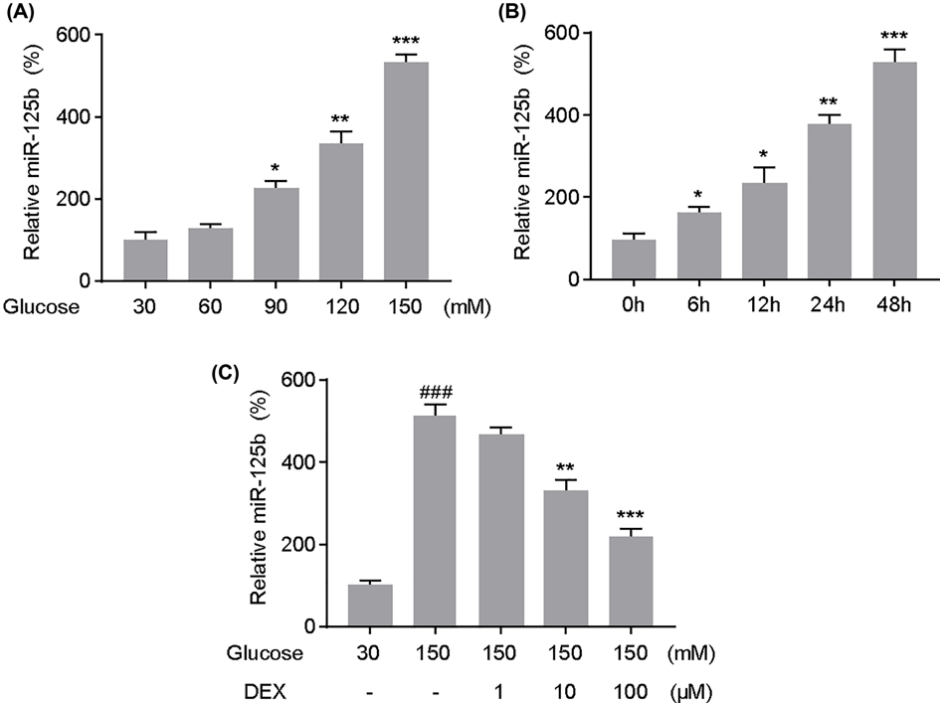
Expression of miR-125b-5p in PC12 cells under high-glucose conditions (**A**) Expression of miR-125b-5p in PC12 cells at different glucose concentrations was quantified via quantitative polymerase chain reaction analysis. (**B**) Expression of miR-125b-5p in PC12 cells at different treatment intervals upon treatment with 150 mM glucose. (**C**) Effects of dexmedetomidine (DEX) on the expression of miR-125b-5p in PC12 cells under high-glucose conditions. MiR-125b-5p expression was quantified after PC12 cells cultured with glucose or co-cultured with DEX for 48 h. ###*P*<0.001 vs. Glucose 30 mM; *P<0.05, **P<0.01, ***P<0.001 vs. Glucose 150 mM. One-way analysis of variance was performed for statistical comparisons. Data are presented as mean ± SEM values, each performed in triplicate.

Equivalent concentrations of mannitol were used as an osmotic control and the results showed that HG had little effect on miR-125b-5p expression (data not shown). qRT-PCR analysis revealed that miR-125b-5p was significantly up-regulated in a dose-dependent manner under high-glucose conditions for 48 h (Figure 3A). When extending the treatment interval, miR-125b-5p was further up-regulated with prolonged time under 150 μM glucose (Figure 3B). However, upon treatment with 10 or 100 μM DEX, miR-125b-5p was markedly down-regulated in PC12 cells when co-cultured with 30 μM glucose for 48 h (Figure 3C).

### Up-regulation of miR-125b-5p reversed the protective effects of DEX against high glucose-induced injury in PC12 cells

**Figure 4:**
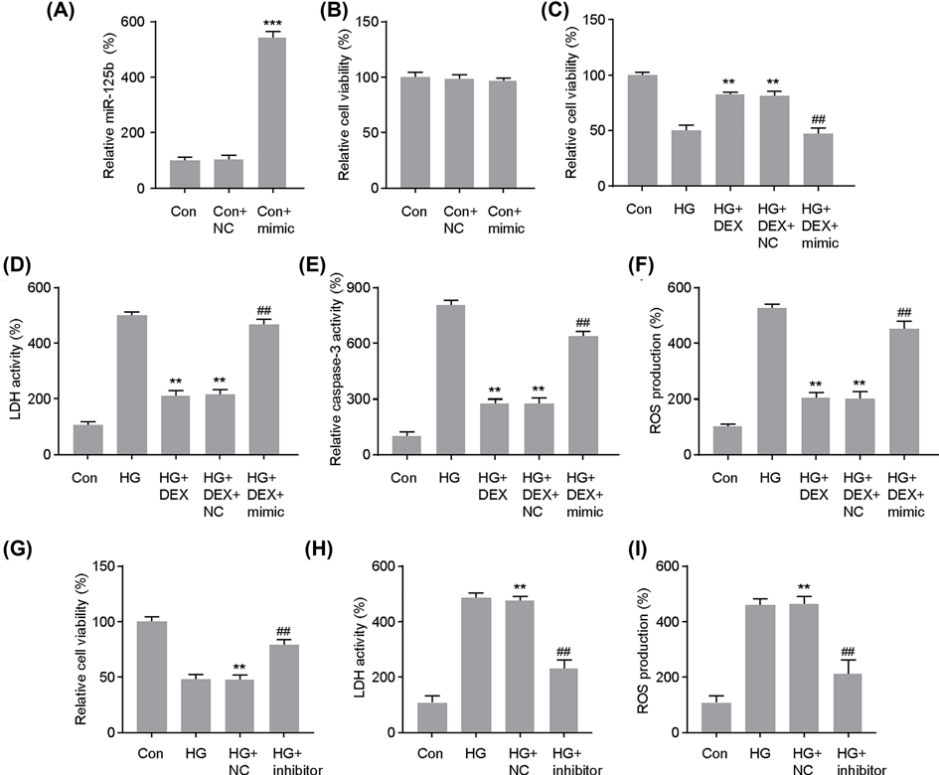
Up-regulation of miR-125b-5p in the course of action of dexmedetomidine (DEX) on the survival and apoptosis in PC12 cells under high-glucose conditions (**A**) Expression of miR-125b-5p in cells transfected with miR-125b-5p mimics or NC; ***P<0.001 vs. Con. (**B**) Cell viability was evaluated using the MTT assay in cells transfected with miR-125b-5p mimics or NC. The effects of the miR-125b-5p mimics transfection on the cell viability (**C**), lactate dehydrogenase activity (**D**), Caspase-3 activity (**E**) and ROS production (**F**) were assessed to evaluate the its influence on the neuroprotective effect of DEX in PC12 cells; **P<0.01 vs. HG, ##*P*<0.01 vs. HG+DEX+NC. The effects of the miR-125b-5p-inhibitor transfection on the cell viability (**G**), lactate dehydrogenase activity (**H**) and ROS production (**I**) were assessed to evaluate the its neuroprotective effect under HG in PC12 cells. **P<0.01 vs. HG, ##*P*<0.01 vs. HG+NC. One-way analysis of variance was performed for statistical comparisons. Data are presented as mean ± SEM values, each performed in triplicate.

To investigate the role of miR-125b-5p in DEX-mediated protection in PC12 cells, miR-125b-5p was overexpressed in PC-12 cells via miRNA transfection. As shown in Figure 4A,B, the transfection of miR-125b-5p-mimic significantly increased the expression level of miR-125b-5p while had little effect on the cell viability. We then further explored whether regulating miR-125b-5p expression influences the protective effect of high glucose-induced injury in PC12 cells. We transfected the miR-125b-5p-mimic into the PC12 cells under HG and DEX conditions. The restoration of miR-125b-5p reversed the increased the viability of PC12 cells mediated by DEX (Figure 4C). As shown in Figure 4D,E, the suppressive effects of DEX on cell injury in PC12 cells cultured under high-glucose conditions were reversed upon miR-125b-5p overexpression. Furthermore, ROS production in PC12 cells was remarkablely increased upon transfection with the miR-125b-5p mimic under high-glucose conditions and DEX treatment (Figure 4F). In addition, we also transfected the PC12 cells with miR-125b-5p-inhibitor to further validate the role of miR-125b-5p in relation to HG and DEX’s neuroprotection effect. We transfected miR-125b-5p inhibitor into the PC12 cells under HG condition and the results showed that it exert similar neuroprotection role with DEX (Figure 4G–I). These results further confirmed the role of DEX in high glucose-induced cell injury and apoptosis and the potential novel therapeutic target for miR-125b-5p.

### VDR is the target of miR-125b-5p

**Figure 5:**
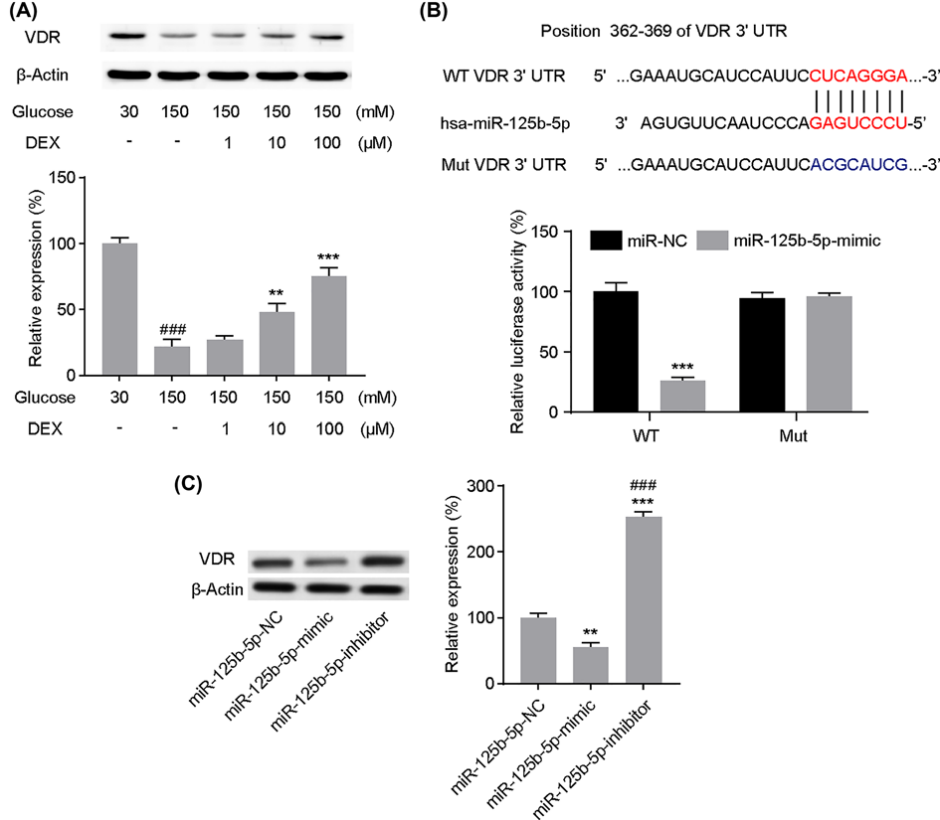
Interactions between miR-125b-5p and the vitamin D receptor (VDR) (**A**) VDR expression in PC12 cells under high-glucose conditions was examined via Western blot analysis. ###*P*<0.001 vs. Glucose 30 mM; **P<0.01, ***P<0.001 vs. Glucose 150 mM. (**B**) Bioinformatic analysis was performed to predict miR-125b-5p binding sites in VDR and luciferase reporter assays were conducted after miR-125b-5p mimic and VDR-WT or VDR-Mut reporter plasmid were co-transfected into PC12 cells. ***P<0.001 vs. miR-NC. (**C**) VDR expression in PC12 cells transfected with the miR-125b-5p mimic or inhibitor was assessed after 24 h of transfection; **P<0.01, ***P<0.001 vs. miR-NC; ###*P*<0.001 vs. miR-NC. One-way analysis of variance was performed in (A and C) and the independent samples *t*-test was performed in (B). Data are presented as mean ± SEM values, each performed in triplicate.

Numerous studies have reported that mRNA exerts its regulatory functions by targeting with downstream regulators. To further reveal the mechanisms underlying the protection on PC12 cells under high glucose-induced cellular damage via miR-125b-5p, we assessed the expression of miR-125b-5p. Relatively lower VDR expression was observed in PC12 cells under high-glucose conditions in comparison with normal cultured cells, while DEX up-regulated VDR (Figure 5A). Equivalent concentrations of mannitol were used as an osmotic control and the results showed that HG had little effect on VDR expression (data not shown). Bioinformatic analysis (http://www.targetscan.org/) revealed a conserved binding site for miR-125b-5p at the 3′-UTR of VDR mRNA (Figure 5B). Then, we performed the dual luciferase reporter assay to confirm whether miR-125b-5p directly interacts with the VDR. VDR binding sites (WT-VDR) or mutant binding sites (Mut-VDR) in miR-125b-5p were cloned into the luciferase vector. Co-transfection of the miR-125b-5p mimic and the WT-VDR luciferase vector dramaticly decreased luciferase activity, while co-transfection of the miR-125b-5p mimic and the Mut-VDR luciferase vector did not significantly influence luciferase activity (Figure 5B). To confirm the association between miR-125b-5p and VDR furtherly, we performed Western blot to detect the direct effect of miR-125b-5p on VDR expression. Relative VDR expression was elevated in PC12 cells transfected with miR-125b-5p inhibitor and significantly reduced upon transfection with the miR-125b-5p mimic (Figure 5C). Together, these results suggest that miR-125b-5p targets the VDR and regulates the expression of VDR in PC12 cells.

### VDR reversed the induction of miR-125b-5p overexpression and apoptosis upon DEX treatment

**Figure 6:**
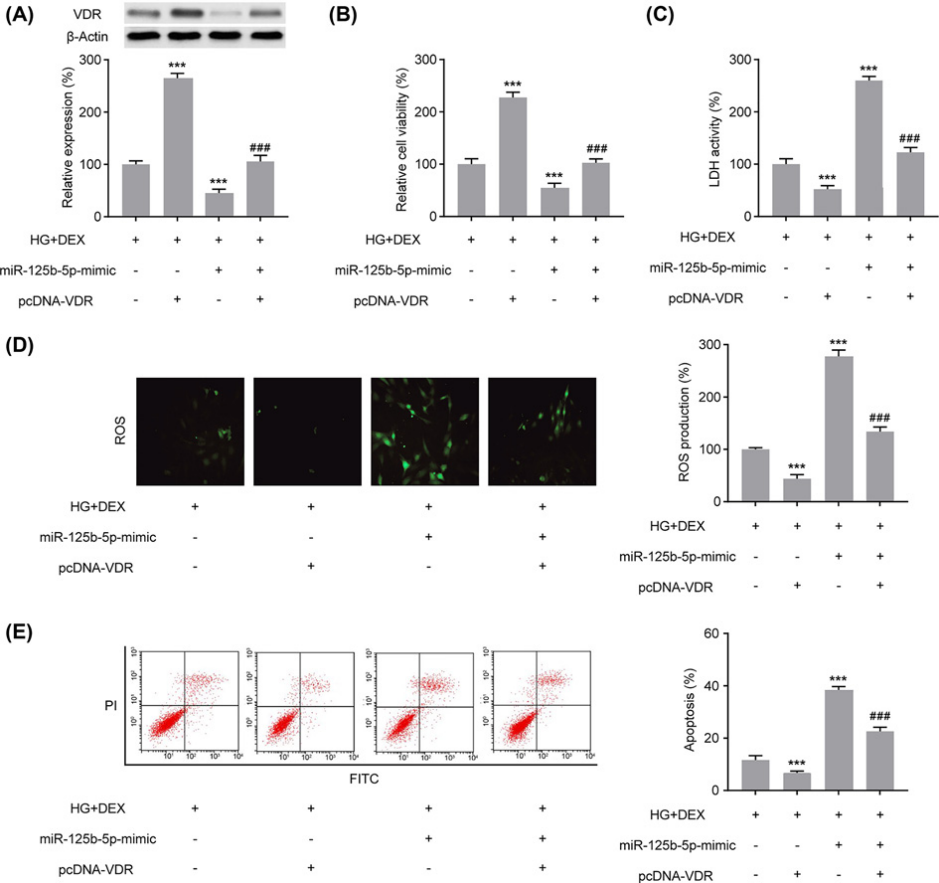
DEX exert its neuroprotective role under high-glucose conditions through regulating the miR-125b-5p/VDR axis in PC12 cells (**A**) VDR expression in PC12 cells after transfected with miR-125b-5p-mimic or pcDNA-VDR under high-glucose conditions and DEX treatment. The effects of the miR-125b-5p-mimic or pcDNA–VDR transfection under high-glucose conditions and DEX treatment on the cell viability (**B**), lactate dehydrogenase activity, (**C**), ROS production (**D**) and cell apoptosis (**E**) were assessed in PC12 cells. After transfection with the miR-125b-5p mimic or pcDNA-VDR for 24 h, PC12 cells were co-cultured with HG and 100 μM DEX for 48 h. One-way analysis of variance was performed. ***P<0.001 vs. HG+DEX+miR-125b-5p-NC+pcDNA-Con, ###*P*<0.001 vs. HG+DEX+-miR-125b-5p-mimic+pcDNA-Con. Data are presented as mean ± SEM values, each performed in triplicate.

To further investigate the regulatory role of VDR in the inhibition of cellular injury via miR-125b-5p, VDR was up-regulated via plasmid transfaction and/or miR-125b-5p was up-regulated via miR-mimic transfaction upon DEX and high-glucose treatment. As shown in Figure 6A, VDR protein expression was induced in PC12 cells upon pcDNA–VDR transfection and significantly suppressed upon transfection with the miR-125b-5p mimic, which validated the effectiveness of pcDNA-VDR vectors. The inhibition effects of miR-125b-5p up-regulation on cell viability were markedly reversed upon VDR overexpression (Figure 6B). Furthermore, pcDNA–VDR decreased LDH activity (Figure 6C), ROS production (Figure 6D) and apoptosis rate (Figure 6E) in PC12 cells in comparison with the transfection with the miR-125b-5p mimic under high-glucose conditions and DEX treatment. These results indicate that miR-125b-5p inhibition protects PC12 cells against high glucose-induced cellular injury and apoptosis after DEX treatment via VDR regulation.

## Discussion

As a highly selective α2-adrenergic receptor agonist, DEX is clinically used as an efficient and adjuvant drug for anesthesia [5]. Numerous recent studies have reported the neuroprotective effect of DEX in various models of brain injury. Marc Schoeler et al. reported that DEX protected traumatically injured hippocampal cells in vitro, which can be reversed upon the administration of the mitogen-activatedprotein kinase (MAPK) pathway MAP kinases specific inhibitor PD98059 [22], while Stefanie Endesfelder et al. reported that DEX may exert neuroprotective effects in a neonatal mouse model of acute hyperoxia [23]. However, the role of DEX in high-glucose induced brain injury is unclear. In the present study, high-glucose treatment was administered to PC12 cells to simulate neuronal injury in vitro. Our results suggest that DEX protects PC12 cells injuries under high glucose by inhibiting ROS accumulation and apoptosis. Furthermore, our results indicate that the mechanism of neuroprotective effects DEX displayed is related with miR-125b-5p up-regulation and modulation of VDR signaling pathways. In our experiments, equivalent concentrations of mannitol were used as an osmotic control. The results showed that different glucose concentrations had little effect on cell viability, neither the mRNA level of miR-125b-5p nor VDR. Therefore, it could be infered thart high-glucose–induced alteration of miR-125b-5p and VDR levels were not associated with osmotic alteration. Our results indicate that DEX is a potentially effective therapeutic approach for preventing DNP.

Four major complications, viz., nephropathy, neuropathy, retinopathy and vasculopathy are associated with the pathogenesis of diabetes mellitus. DNP is clinically divided into asymmetric and symmetric forms [24]. High-glucose conditions are responsible for neural dysfunction, neuronal injury and apoptosis. As a sedative and pre-anesthetic medication, the side effects of DEX includes peripheral bradycardia, vasoconstriction and decreased cardiac output. It also has been reported that DEX has effects on the endocrine system and glucose homeostasis [25]. Significantly decrease of plasma insulin concentration and increase of the plasma glucose concentration were observed in dogs after DEX treatment [26]. However, whether DEX play a role in high-glucose injury, especially in nervous system is unclear. Liu et al. reported that DEX protects high-glucose induced apoptosis in human retinal pigment epithelial cells, which indicates the promising effects of DEX on high-glucose injury [27]. In the present study, we found that high-glucose conditions resulted in cytotoxicity in PC-12 cells, and cell viability decreased with an increase in LDH release and caspase-3 activity, while DEX treatment reversed cellular injury and apoptosis in PC12 cells, as revealed through flow cytometry analysis. Various mechanisms reportedly underlie hyperglycemia-induced neuronal dysfunction and injury, one of which is oxidative stress. In the present study, ROS level was increased in high glucose-treated PC12 cells, and DEX treatment reduced oxidative stress. These results indicate the neuroprotective effects of DEX under high-glucose conditions.

Recently, miRNAs have received increasing attention owing to their role in different diabetes-related complications. MiR-146a and miR-128a have been reported to be associated with the susceptibility to diabetic polyneuropathy; miR-146a and miR-27a, the susceptibility to cardiovascular autonomic neuropathy [28]. A recent study involving miRNA sequencing reported that miR-125b-5p is closely associated with neuropathic pain. However, limited information is available regarding the role of miR-125b-5p in DNP regulation. In the present study, miR-125b-5p was up-regulated with an increase in glucose concentration and processing time, while DEX treatment reversed miR-125b-5p up-regulation under high-glucose conditions. MiR-125b-5p plays a critical role in cell survival and apoptosis. MiR-125b-5p is involved in the progression of various cancers and inhibits apoptosis as a tumor suppressor [29]. Hua et al. reported that miR-125b-5p overexpression inhibits cell proliferation and metastasis in hepatocellular carcinoma [30]. In the present study, we investigated the effect of miR-125b-5p on the PC12 cells growth under high-glucose conditions. The present results show that miR-125b-5p overexpression reversed the protective effects of DEX on PC12 cells under high-glucose conditions by decreasing cell proliferation, injuries and apoptosis. These findings suggest that DEX potentially interacts with miR-125b-5p to protect against high glucose-induced neuronal injury. Since high glucose-induced neuronal injury contributes to DNP pathogenesis, miR-125b-5p may also play a critical role in DNP pathogenesis. Conversely, Xiong et al. reported that it is miR-125b-5p inhibitor could rescue the sevoflurane-induced hippocampal apoptosis and inflammation in rats. Moreover, the miR-125b-5p was observed to up-regulate in both sevoflurane–anesthesia rats and sevoflurane-treated SH-SY5Y cells [20]. The role of miR-125b-5p in different anesthetic-induced nerve cell apoptosis needs further study. And whether glucose is the key reason of the difference of miR-125b-5p function is a question of thought.

The VDR is a superfamily of nuclear hormone receptors interacting with vitamin D. Vitamin D and VDR regulate multiple pathophysiological pathways, including calcium phosphorus metabolism, anti-inflammation, and cancer prevention, proliferation and differentiation [31]. Epidemiological studies have reported that high serum vitamin D levels are involved with a low diabetes risk [32,33]. Furthermore, vitamin D deficiency is potentially associated with DNP and insulin resistance during pregnancy [34]. Our results show that VDR is a potential target of miR-125b-5p. The biological effects of vitamin D and VDR regulate multiple pathophysiological pathways, including immune regulation, anti-inflammation and anti-infection [35]. In Type 1 diabetes (T1DM), insulin secretion is potentially impaired owing to a vitamin D deficiency and is improved through dietary vitamin D repletion [36], while a lower vitamin D intake or lower 25(OH)D levels might increase the Type 2 diabetes risk [35]. VDR is expressed in the cells of intestinal epithelium, mammary epithelium, renal tubules, pancreas, pituitary gland, etc. [37]. Recent studies have reported the association between vitamin D deficiency and DNP. VDR is up-regulated in T1DM with mild neuropathy and further up-regulated in T1DM with severe neuropathy in comparison with the epidermis, stratum basale, stratum spinosum, overall microvessels, endothelium and pericytes [38]. However, the relationship between and miR-125b-5p and VDR in high glucose injury has not been reported. Our results show that inhibition of miR-125b-5p up-regulated VDR, which further attenuated ROS accumulation induced by high glucose and apoptosis in PC12 cells and reversed the effects of miR-125b-5p overexpression-induced neuronal injury. Therefore, high glucose-induced miR-125b-5p expression potentially inhibits VDR expression, leading to neuronal damage in DNP.

Our study report the effectiveness of DEX in high glucose-induced neuronal injury and the potential relevance of miR-125b-5p and VDR in DNP pathogenesis. Well, we had to confess that our *in vitro* HG model could only partly mimic the pathogenesis of diabetic neuropathy, this is the limition of our study. For future studies, we propose to investigate the neuroprotective effects of DEX in cultured neural cells as well as *in vivo* animal model of diabetic neuropathy are essential to investigate the protective effects of DEX and the precise role of miR-125b-5p in DNP regulation. Overall, the present study shows that DEX plays a neuroprotective role in high glucose-induced neuronal injury by inhibiting miR-125b-5p expression and activating VDR expression. DEX and other agents targeting the miR-125b-5p /VDR pathway are potentially applicable in DNP treatment. Due to microRNA molecules are very stable in serum, our research also provides a new biomarker for the diagnosis of DNP.

## Abbreviations

CVD: cardiovascular disease
DEX: dexmedetomidine
DNP: diabetic neuropathy
HG: high glucose
LDH: lactate dehydrogenase
MTT: 3-(4,5-dimethylthiazol-2-yl)-2,5-diphenyltetrazolium bromide
T2DM: Type 2 diabetes
VDR: vitamin D receptor
